# Rheumatic Digital Twin: Proposed Machine Learning–Based Multimodal Framework to Inform Clinical Decision-Making

**DOI:** 10.2196/86763

**Published:** 2026-05-04

**Authors:** Daniyal Selani, Rachel Knevel, Marcel Reinders, Erik B van den Akker

**Affiliations:** 1 Pattern Recognition and Bioinformatics, Faculty Electrical Engineering, Mathematics and Computer Science, Delft University of Technology, Delft, The Netherlands; 2 Department of Rheumatology, Leiden University Medical Center, Leiden, The Netherlands; 3 Department of Biomedical Data Sciences, Leiden University Medical Center, Einthovenweg 20, Leiden, 2333 ZC, The Netherlands, 31 1 526 30 13

**Keywords:** digital twin, artificial intelligence, AI, rheumatology, electronic health records, machine learning, multimodal data

## Abstract

Rheumatic diseases are chronic, immune-mediated conditions characterized by significant heterogeneity in presentation and disease course. However, current clinical approaches often rely on snapshot-based assessments that fail to capture the complex longitudinal evolution of these conditions. To address these limitations and support the implementation of precision medicine, we present the design for the Rheumatic Digital Twin, a novel, modular conceptual framework intended to integrate heterogeneous multimodal data, ranging from electronic health records and clinical notes to imaging and omics, into a dynamic, computational representation of the patient journey. Our theoretical architecture addresses challenges related to data silos and variable availability of data modalities through a multistage approach that envisions the use of domain-specific foundation models to independently process distinct data modalities. To effectively model the temporal progression inherent in chronic diseases, the proposed design utilizes Transformer architectures, leveraging self-attention mechanisms to treat patient events, such as lab results or medication changes, as sequential data tokens. We describe how these unimodal representations would subsequently be fused via joint embedding techniques to construct a shared, multimodal representational space. Envisioned to function analogously to a recommender system, the Rheumatic Digital Twin framework is modeled to map patients into a latent space where proximity reflects clinical and biological similarity. By identifying “nearest neighbors,” historical patients with comparable trajectories, the system aims to enable in silico cohorting, theoretically allowing clinicians to forecast key clinical events, predict treatment responses, and identify likely disease courses based on the outcomes of similar peers.

## Introduction

Rheumatic diseases (RDs) are chronic, immune-mediated conditions marked by persistent inflammation affecting joints, connective tissues, and internal organs. RDs are progressive, often disabling, and notoriously heterogeneous in both presentation and disease course. Early diagnosis and individualized treatment are essential to improving outcomes and preventing irreversible damage, yet current clinical approaches remain ill-equipped to handle the complexity of these diseases [1]. Traditional RD classification and diagnostic frameworks rely on clinical assessment, patient-reported symptoms, imaging, and blood-based biomarkers. While valuable, these sources often offer limited resolution, capturing only fragments of a patient’s disease profile. Crucially, they fail to reflect the underlying molecular mechanisms and individualized risk factors that drive disease heterogeneity. Collectively, the current classification leads to patient groupings that appear clinically similar but may differ substantially in their biology, prognosis, and treatment response.

To address these limitations, researchers have turned to multimodal data integration, combining clinical variables with genetic, transcriptomic, proteomic, and imaging data [2]. These efforts have shown promise; for example, transcriptomic profiling has been used to define novel inflammatory endotypes in rheumatoid arthritis (RA) [3], and combined clinical-genetic models have improved flare prediction [4]. Yet, most approaches remain constrained by siloed data analysis, one-time snapshots, and limited longitudinal context. The full potential of multimodal data in rheumatology has yet to be realized.

We argue that a more comprehensive, dynamic, and patient-specific model is needed; one that evolves with the patient and enables interpretation within the broader disease context. This is where the concept of the medical digital twin (MDT) becomes particularly compelling. A digital twin (DT) is a continuously updating computational representation of an individual patient that utilizes data-driven models to integrate diverse data streams over time, ranging from electronic health records (EHR) and omics profiles to imaging, wearables, and lifestyle data [5]. MDTs have already demonstrated promise in oncology [6], cardiology, and critical care [7], enabling simulation of disease progression, real-time risk prediction, and adaptive therapy planning. The application of MDTs within rheumatology, however, is still in its infancy [8]. Existing MDT approaches often fall short when it comes to effectively integrating high-dimensional, multimodal data into a unified representation of the patient [9]. This integration is critical; the utility of an MDT depends not just on data accumulation but on mapping these data modalities into a shared space in which features of a patient can be aligned and compared meaningfully with those of previously admitted patients.

In response to these challenges, this paper presents the conceptual design and architectural specifications for the Rheumatic Digital Twin (RDT). The primary novelty of our work lies in the proposal of an architecture specifically engineered to address the unique complexities of RDs, which are characterized by an exceptional degree of multimodal heterogeneity and long-term, fluctuating disease courses. Unlike existing models that often rely on static data snapshots, the proposed RDT architecture introduces a modular, Transformer-based fusion framework designed to capture the dynamic, temporal evolution of the patient’s journey.

The contribution of the proposed RDT framework is threefold. First, it enables the integration of diverse, high-dimensional data into a unified representational space. Second, the modular design allows for independent handling of each modality, making the system resilient to missing data and scalable for clinical environments with varying resources. Finally, by positioning individual patient journeys within a broader population context, the RDT facilitates “in silico cohorting,” allowing clinicians to forecast outcomes and treatment responses by analogy with similar historical cases. This application-specific approach provides a necessary foundation for moving beyond traditional RD classification toward a truly personalized, data-driven model of care.

While the RDT framework is applicable across the spectrum of rheumatology, it is particularly positioned to benefit complex, heterogeneous conditions such as RA, systemic lupus erythematosus, and systemic sclerosis. In these diseases, clinical presentation often diverges significantly from underlying molecular pathology; patients may share similar symptoms but follow vastly different trajectories regarding joint erosion, renal involvement, or interstitial lung disease. By integrating multimodal streams ranging from ultrasound synovitis scores to transcriptomic profiles, the RDT aims to better resolve these diverse endotypes than traditional clinical classification criteria.

## Recommender Systems as DTs: An Intuitive Definition

There is currently no broad consensus on the definition of DTs in general, let alone for applications in the medical domain. In their most ambitious implementations, DTs aim to exhaustively model all aspects of a system [10]. However, demands on the available data are often limiting, thus creating implementation boundaries. In the medical context, these limitations involve the variety, the variable availability, and the variable sampling frequencies of data types measured for patients.

We define MDTs as an information system or a collection of systems capable of integrating patient data aggregated during the patient journey to generate patient representations that are clinically actionable and predictive of outcomes, useful for supporting clinical decision-making tasks. As our MDT is rooted in the longitudinal sequence of events, it constructs a DT of the patient’s journey, treating the evolving clinical narrative as the defining feature of the patient’s representation. This is particularly important in our multimodal approach, where each event can encompass data from various modalities (eg, lab results, imaging, clinical notes).

It is important to acknowledge that the pursuit of a full-scale MDT exists alongside a prevailing clinical preference for simpler, established predictive models. In many practical settings, clinicians may prioritize targeted omics studies or validated “snapshot” biomarkers because they offer high interpretability and lower resource requirements compared to the inherent complexity of a DT. These simpler models are often easier to implement within existing time-sensitive workflows and provide clear, actionable evidence for specific clinical questions. However, we argue that while these traditional approaches are relevant, they are often ill-equipped to handle the deep heterogeneity and chronic, evolving nature of RDs. Relying solely on static, unidimensional analysis risks missing the latent disease states and emergent phenotypes that only a dynamic, multimodal representation can reveal. The RDT is not intended to replace these tools; in fact, it ingests exactly these mutually complementary data points to comprehensively capture the whole patient journey, thereby aiming to provide a level of precision that single-instance models cannot achieve.

Our proposed MDT seeks to embed patient data in a space where patient similarities and differences are amplified. Similar patients are mapped close together, whereas dissimilar patients are mapped further apart. This concept is analogous to how recommender systems for services such as Amazon and Netflix work [11]. Even though these are surface-level similarities, the task of recommending movies or shopping items based on previous customers with very similar preferences and interests provides an intuitive way to understand our DT proposal. For instance, in our MDT, “shop items” or “movies” become “treatment options,” and “customer preferences and interests” are replaced by all recorded interactions between the patient and the health care system, that is, the multimodal patient data. Ochoa et al [12] have already shown how a recommendation system–like approach can be utilized for treatment decision support.

Recommender systems for commercial services typically define a space in which users with similar interests are closely aligned, effectively mapping similar users into the same neighborhoods or clusters using embedding methods or clustering algorithms. The underlying assumption is that users who exhibit similar behavior, recorded via interactions with the website, will have similar interests and, hence, would want to buy or watch similar items. Interestingly, these systems also track user behavior over time to account for changes in taste or interest. This temporal aspect is equally crucial for applications in the medical domain, where patient journeys unfold over time, and incorporating time-resolved multimodal data allows us to capture the evolving nature of the patient’s condition in high detail [5].

Drawing parallels, our hypothesis is that patients with closely resembling phenotypic, genetic, demographic, and clinical traits may have similar disease states and could, therefore, respond to similar treatments. That is, patients who have some underlying similarities based on the complex interaction of factors might develop similar or identical disease states. This is particularly relevant when considering multimodal data, as the “interaction of factors” can involve complex relationships among genetic predispositions, lifestyle choices, and various clinical measurements. This is useful because we can use existing data from previous patients to stratify new patients into clinically relevant clusters and trajectories. Placing individual patients within such multimodal representational spaces that encapsulate the full heterogeneity of the broader RD population unlocks powerful possibilities for personalized medicine and translational insight. Patients positioned near each other in this space share not just superficial clinical features but deeper molecular and phenotypic similarities. This contextualization allows clinicians and researchers to infer likely disease trajectories, therapeutic responses, and even comorbidity risks by analogy with similar patients, essentially enabling in silico cohorting.

For instance, if a newly diagnosed patient maps closely to a cluster of individuals who responded favorably to a specific biologic therapy, this evidence can inform treatment selection, even in the absence of extensive prior clinical history. Beyond treatment prediction, this context also allows for early identification of at-risk patients, continuous monitoring for deviations from expected patterns, and hypothesis generation for underexplored subgroups. Moreover, the evolving structure of the patient space itself can reveal latent disease states, emergent phenotypes, or signatures of response that are not evident through unidimensional analysis. In this way, DTs not only support individual patient care but also serve as a foundation for continuous learning and discovery within the rheumatology domain.

## A Modular Multimodal MDT

**Figure 1.**
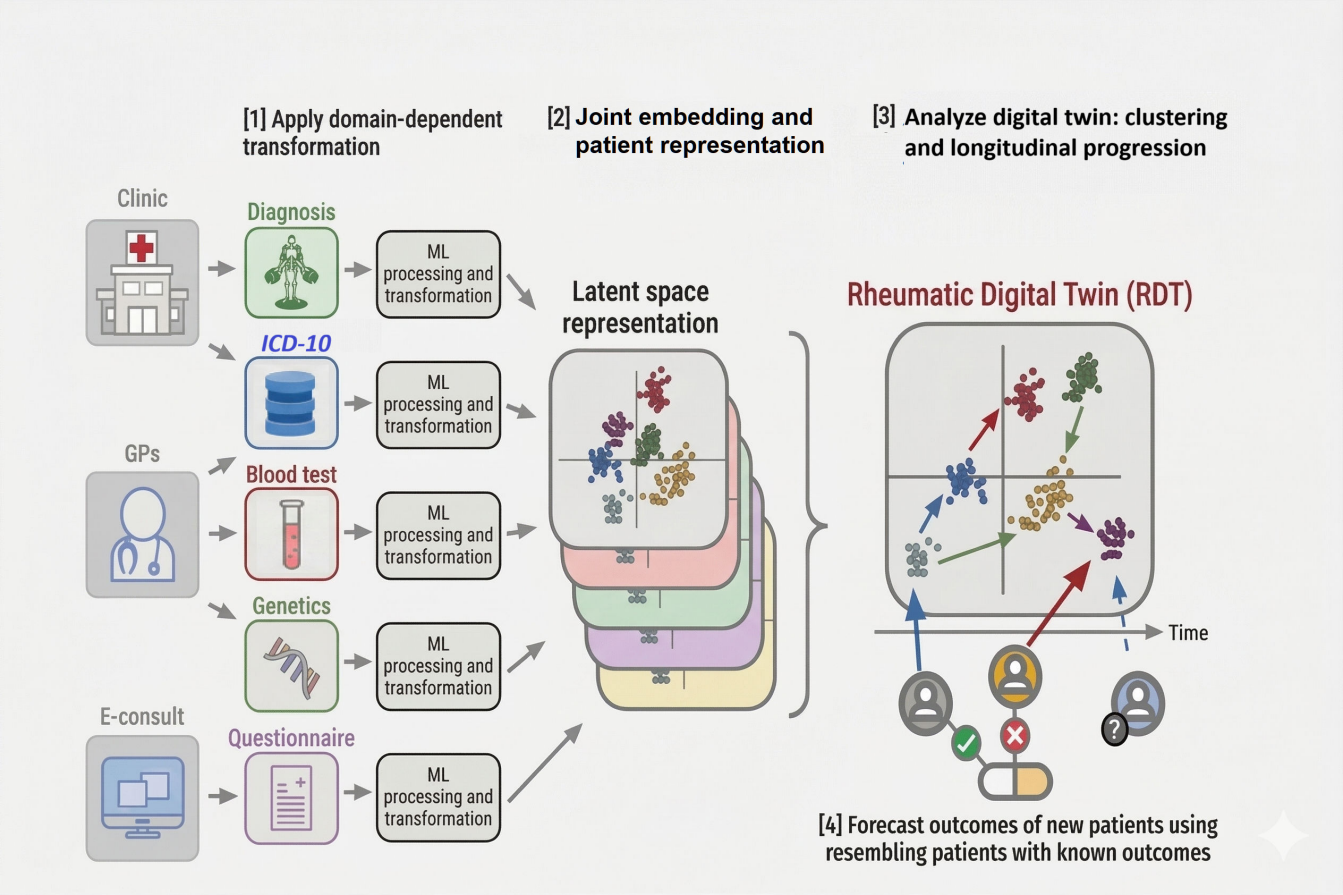
Rheumatic Digital Twin (RDT). Architecture of the multimodal RDT for precision patient stratification and outcome forecasting. (1) Diverse clinical and biomedical data sources, including physical assessments, billing codes (in the *International Classification of Diseases, 10th Revision* [*ICD-10*] standard), blood tests, and, optionally, genetic profiles and patient questionnaires, undergo domain-specific transformations. (2) These reduced representations are integrated through joint embedding to construct a patient-patient network that captures interpatient similarity across modalities. (3) The resulting representations enable unsupervised clustering to uncover disease subtypes. By analyzing these representations longitudinally, the model captures the evolving “patient journey” through distinct states, building on the concept of “cluster journeys” [13,14] to define treatment response profiles and relapse trajectories over time. (4) New patients are mapped onto this latent space to forecast outcomes by identifying their nearest neighbors among patients with known trajectories. GPs: general practitioners; ML: machine learning.

Given the practical constraints of data availability and the varying nature of health care data, we propose a flexible architecture that can adapt to diverse tasks and data types. In clinical settings, data modalities can vary significantly in their ease of collection, granularity, and frequency. For example, questionnaire data can be easily collected for many patients, while genomic data is often restricted by high costs, technical expertise, and privacy concerns. The architecture of our proposed MDT (Figure 1) is designed to be modular, allowing for the integration of different types of data depending on the specific needs of the task at hand and the resources available. Instead of developing a single model that tries to process and integrate all available data types simultaneously, we propose the independent handling of each data modality (Figure 1, panel 1), which can then be combined into a joint embedding (Figure 1, panel 2). This joint embedding, once generated, can be integrated into a task-specific pipeline that allows the system to perform specific clinical tasks, such as clustering patients based on phenotypic similarities (Figure 1, panel 3) or predicting treatment responses (Figure 1, panel 4).

**Figure 2.**
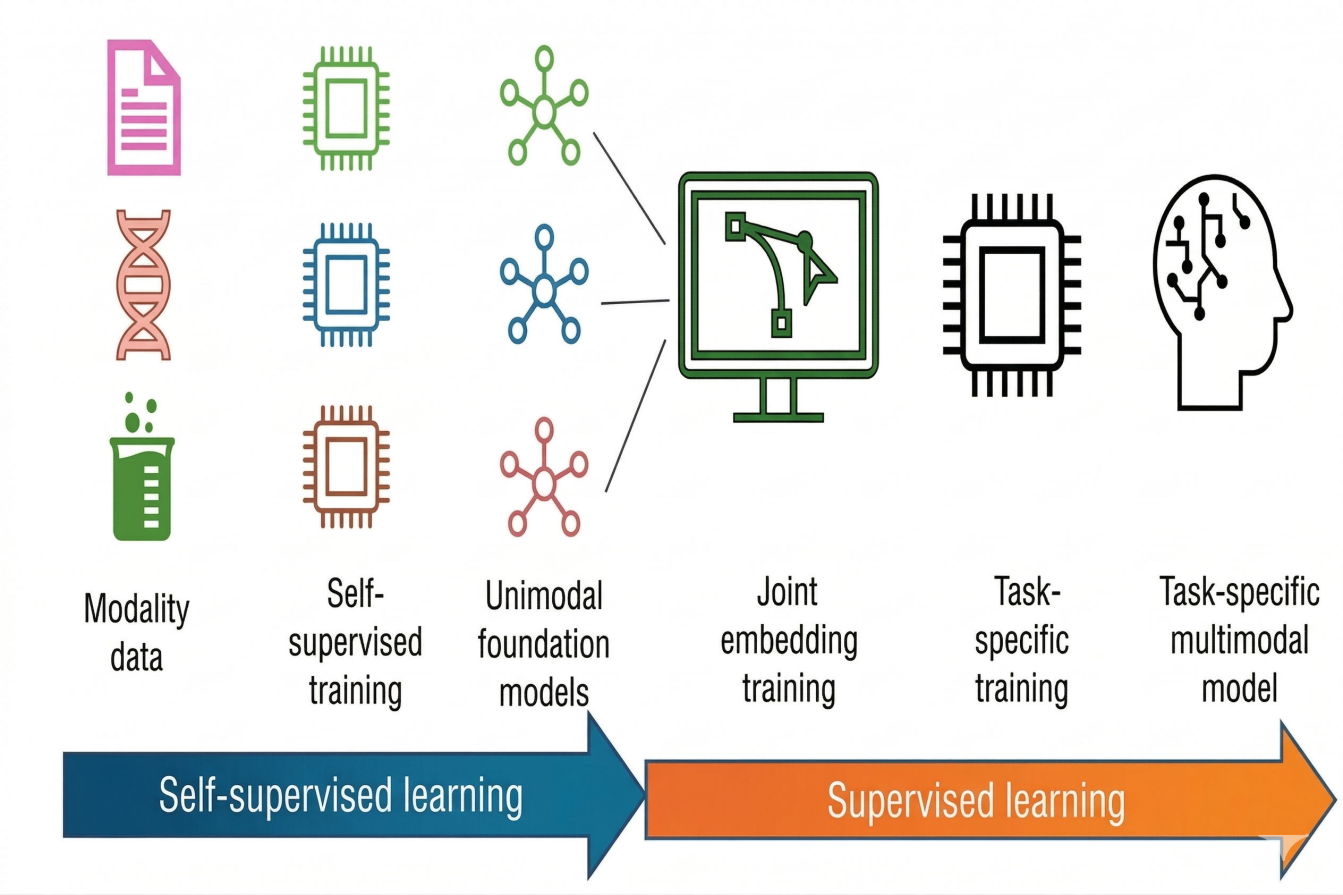
Schematic overview of the multistage workflow for building task-specific multimodal digital twins. Modality-specific data undergoes self-supervised pretraining to yield unimodal foundation models. These models are aligned through a joint embedding training phase, followed by task-specific fine-tuning, culminating in a specialized multimodal model tailored to the target application.

For the embedding of each modality (eg, clinical data, genetic data, imaging, etc), we leverage the concept of foundation models, that is, neural networks trained in an unsupervised manner on large modality-specific datasets to extract meaningful embeddings [15]. These models are trained independently for each modality to preserve their domain-specific characteristics. To combine the individual embeddings into a unified representation, we draw on recent advances in multimodal learning, such as unsupervised contrastive learning [16] and supervised joint embedding models [17] (Figure 2).

Once a patient’s data is embedded into the shared space, it can evolve over time. As new events in a patient’s journey, such as a change in disease activity score or the addition of a new medication, are recorded, the patient’s embedding can be updated. The integration process does not require the entire model to be retrained. Instead, new events can be incorporated by updating the patient’s representation in the embedding space and running the pipeline again. This creates a dynamic 2-way flow of information: decisions informed by the MDT affect the patient’s journey, while new events in the patient’s journey further inform the MDT. The resulting patient embeddings can then be used as inputs for additional models designed to predict disease trajectories, treatment outcomes, or potential complications.

The modular design enhances the system’s flexibility and scalability, as it allows for the addition of new data modalities or tasks without requiring significant reconfiguration of the entire system. For example, the system can initially be developed using readily available clinical data and later be expanded to include genomic or imaging data as these modalities become more accessible. This also enables continuous refinement of the MDT system, which can evolve alongside advancements in medical technology and the availability of new data sources. As the system grows and more modalities are incorporated, the resulting MDT becomes an increasingly robust tool for providing personalized clinical recommendations.

## Modeling Single Modalities

In chronic diseases, patient care unfolds over extended periods, during which patients’ characteristics, disease states, symptoms, and treatment responses evolve. This dynamic nature of the patient journey necessitates a method of data modeling that can capture the progression of the disease over time and the interaction between past events and future outcomes. Unlike static datasets, longitudinal patient data must reflect not only the sequence of events but also the way each event influences subsequent events, incorporating both medical history and external factors. This ongoing temporal progression means that patients cannot be modeled as isolated snapshots; instead, their health status should be seen as part of an evolving narrative, a “patient journey” that spans multiple visits, treatments, and changes in condition.

To effectively model this continuous and evolving process, we propose using Transformers, a deep learning architecture particularly well suited for sequential data [18]. Transformers are not only capable of modeling complex sequential data but can also learn intricate dependencies across long temporal sequences, making them ideal for capturing the unfolding nature of patient data. Transformers are the backbone of state-of-the-art models in natural language processing because of their ability to handle long-range dependencies, contextual relationships, and the interdependencies between elements within a sequence. In the context of medical data, these “elements” are patient events, such as appointments, treatments, lab results, and disease activity scores. Each of these events can be seen as part of a larger, context-dependent journey, and Transformers excel at modeling these relationships.

At the core of Transformers lies the self-attention mechanism, which is essential for processing sequences with complex interdependencies. The self-attention mechanism works by computing a weighted sum of all elements in the sequence, where the weights reflect the relevance of each element (or event) to others within the sequence. This is crucial when modeling patient journeys, as a single medical event, such as a change in lab results or a prescribed medication, can significantly impact the patient’s subsequent health status. The self-attention mechanism enables the model to prioritize the most relevant events in a patient’s history when making predictions or recommendations, allowing it to focus on the parts of the journey that are most predictive of future outcomes. For example, a previous diagnosis of a comorbidity may strongly influence treatment decisions for a current condition. Transformers can automatically assign a higher weight to such events when making predictions about treatment efficacy or disease progression. The specific definition of an event depends on the modality being modeled. For example, if we are modeling clinical visits, each event might correspond to a single visit, with all associated information, such as diagnoses, treatments, and prescriptions. If we are modeling disease activity scores, each event could represent a new score recorded during a visit or a specific time point in the disease progression.

By treating these patient events as tokens in a sequence, we can apply the same Transformer architecture that has proven successful in natural language processing tasks like machine translation or sentiment analysis. The key to this approach is the self-supervised training method known as masked language modeling. In masked language modeling, portions of the input data are randomly masked, and the model is tasked with predicting the missing information based on the surrounding context. This pretraining strategy encourages the model to learn a deep understanding of the relationships between different events in the patient journey, helping it generate meaningful representations of the patient’s health history. For instance, Zhang et al [7] demonstrated the use of Transformers in modeling patient journeys represented by International Classification of Diseases, 10th Revision (*ICD-10)* billing codes, where the sequence of medical visits and associated codes was treated as events. This approach enabled the Transformer to develop clinically relevant representations of patients, which could then be fine-tuned for tasks such as predicting future disease progression or treatment responses. Similarly, by adopting a Transformer-based model for patient data, we can ensure that each patient’s journey is captured in a rich, contextualized manner, accounting for the temporal relationships between events.

To effectively process the inherent complexity and irregular sampling of EHRs, we employ a specialized tokenization strategy that converts heterogeneous medical data into a standardized sequence for the Transformer. Since rheumatic patient data is often irregularly sampled, we address temporal sparsity by explicitly modeling the time elapsed between clinical encounters. This is achieved by incorporating “gap event” tokens or time-delta embeddings into the sequence, which allows the model to differentiate between events occurring in rapid succession and those separated by months or years. This ensures that the Transformer incorporates the duration between observations as a primary feature, preserving the temporal context of the “evolving narrative” of the patient’s journey.

While alternative architectures, such as temporal convolutional networks [19] or recurrent neural network (RNN) variants like gated recurrent unit with decay [20], are commonly used for sequential data, we prioritize Transformers due to their superior ability to handle long-range dependencies and complex contextual relationships [21]. Unlike strictly recurrent models, which may suffer from vanishing gradients over the multi-year timelines typical of RD [22,23], the self-attention mechanism allows the RDT to prioritize specific historical events, such as a distant comorbidity, that remain highly predictive of current treatment outcomes.

**Figure 3.**
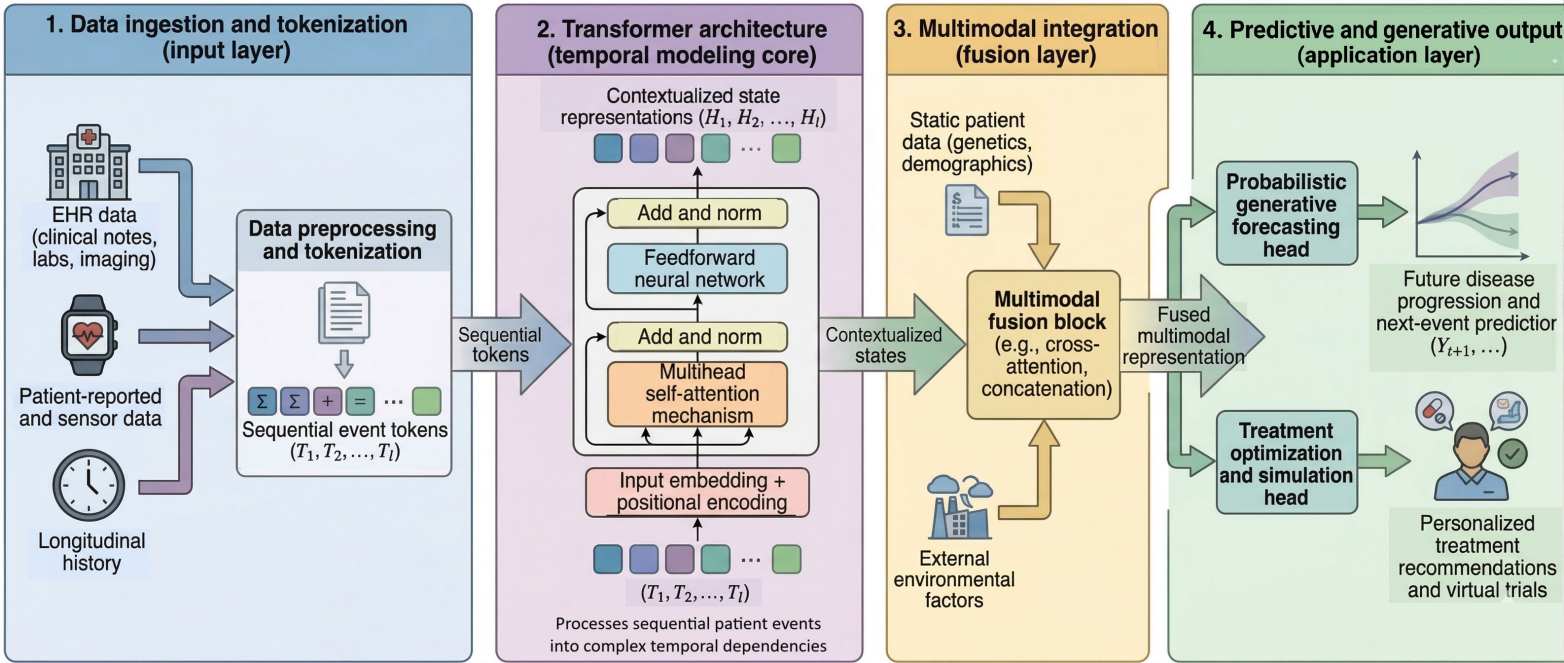
Transformer integration within the Rheumatic Digital Twin framework. The schematic illustrates the model hierarchy, where the Transformer acts as the central temporal core. It processes heterogeneous patient data as sequential tokens (left) to generate contextualized embeddings (center), which are then used by downstream predictive heads for forecasting and treatment simulation (right). EHR: electronic health record.

The conversion of specific data types into tokens is modality-dependent. Structured clinical data, such as *ICD-10* billing codes and specific diagnoses, are treated as discrete tokens similar to words in a vocabulary. For continuous variables, such as irregularly sampled disease activity scores or laboratory values, we either discretize the values into bins or use linear layers to project the raw numerical data into a high-dimensional embedding space. Unstructured data, such as clinical notes from e-consults or physical assessments, are processed via hierarchical tokenization. In this approach, a specialized model like Clinical BERT (Bidirectional Encoder Representations from Transformers) generates a single vector for an entire note. To address the token limits of traditional architectures, we leverage current state-of-the-art methods such as Longformers [24] and sparse attention models [25]. These advancements, driven by improved hardware capabilities, support greatly increased context windows, effectively mitigating truncation issues and ensuring that even lengthy clinical narratives are captured before entering the central Transformer as a single “event token.” By standardizing these diverse inputs into a unified token stream, the Transformer can leverage its self-attention mechanism to identify dependencies between disparate events, regardless of their original format (Figure 3).

## Joint Embeddings

To integrate these diverse perspectives captured by different data modalities, our framework utilizes an intermediate fusion strategy. Rather than aligning data at the raw input level, which would be highly sensitive to noise and formatting differences, each modality is independently processed by its respective foundation model to generate domain-specific embeddings. These embeddings are then projected into a shared latent space via a multimodal Transformer.

A significant challenge in this phase is the inherent “complete missingness” of high-dimensional data, such as genetic or imaging profiles, which may not be available for every patient. To maintain alignment despite these gaps, we employ a self-attention masking mechanism. In this approach, missing modality embeddings are represented as masked or zero-padded tokens before being fed into the joint Transformer. The self-attention mechanism is specifically configured to ignore these masked tokens, ensuring that the learned cross-modal relationships are derived solely from available data. This allows the model to remain resilient; it can still compute a robust patient representation by leveraging the correlations among whatever subset of modalities is present. By treating each modality as an independent but relatable “token” in the patient’s profile, the architecture avoids the rigid requirement of complete data matrices common in traditional multivariate analysis.

To ensure that the joint embedding space preserves meaningful cross-modal relationships rather than merely concatenating data, we propose 2 primary self-supervised learning objectives. First, we use contrastive learning, which is designed to minimize the distance between embeddings of semantically related instances, such as a specific diagnostic imaging feature and its corresponding clinical description, while distinguishing them from dissimilar instances [26,27]. Second, we incorporate a cross-modal masked modeling strategy, wherein portions of one modality (eg, specific lab results) are hidden, requiring the model to reconstruct the missing information using context from available modalities (eg, longitudinal clinical notes) [28,29]. By training the model to predict across different data types, the system is designed to capture the underlying biological and clinical correlations between disparate sources [30,31]. This alignment can enable the RDT to map patients with similar underlying disease mechanisms to the same neighborhood, maintaining robustness even when data profiles are incomplete or derived from differing modalities [17]. These shared embeddings can then be used for tasks such as cross-modal retrieval, where a query image could return the most relevant medical records, or for transfer learning to apply knowledge gained from one data modality to another, less well-studied modality [32,33].

### Clinical Decision Making

Once a patient’s data has been embedded into the multimodal MDT space, the core question becomes: “How can this representation be used to inform clinical decisions?” While embeddings offer a powerful means of integrating diverse sources of information into a unified framework, they are not the end goal. They must be translated into actionable insights. Depending on the clinical use case and the nature of the available data, there are several principled ways in which predictions can be made from this space.

**Figure 4.**
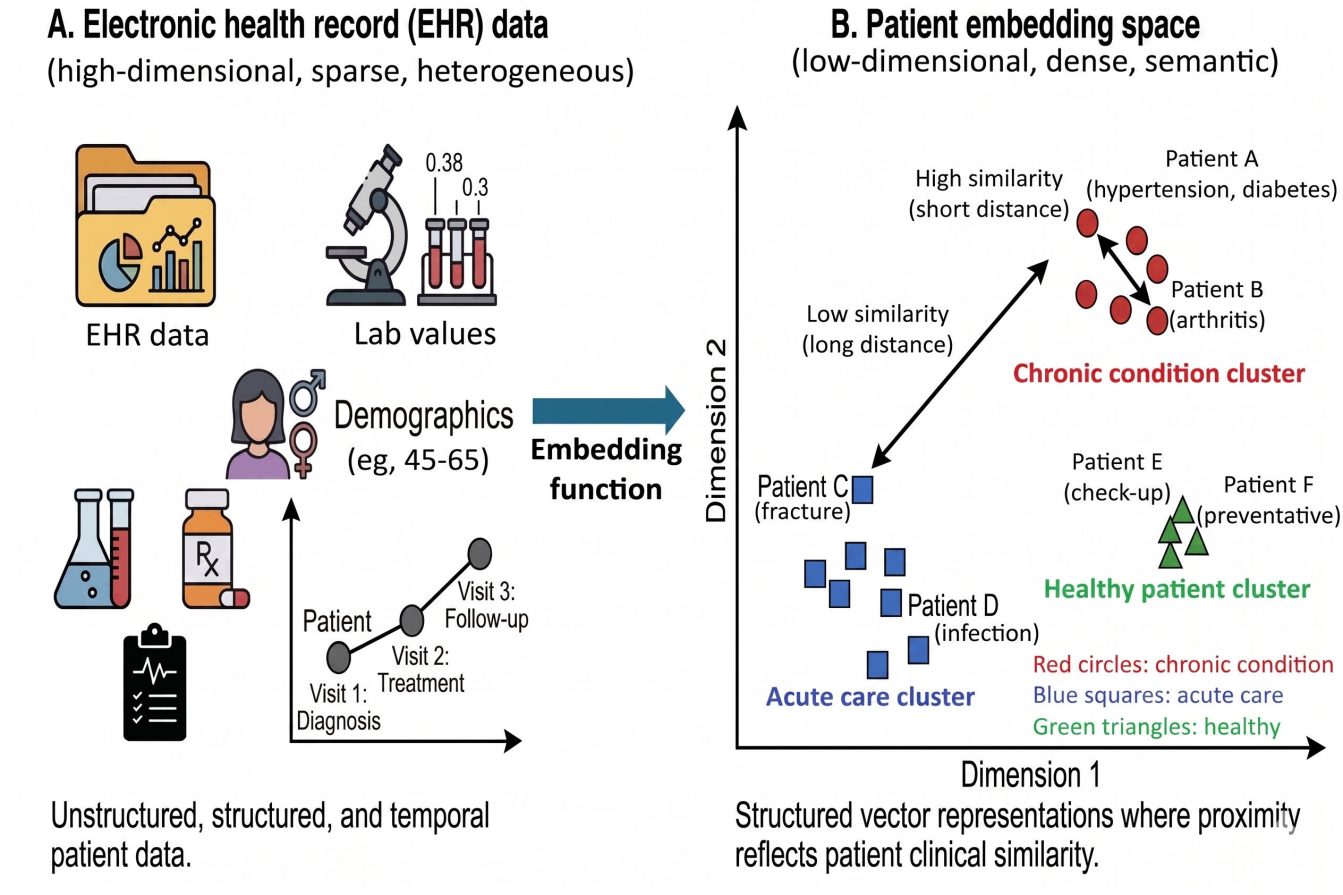
Conceptual illustration of the patient embedding process. (A) High-dimensional input: raw EHR data are characterized as high-dimensional, sparse, and heterogeneous, comprising diverse inputs such as unstructured notes, laboratory values, demographics, and longitudinal visit timelines. (B) Latent embedding space: the embedding function transforms these inputs into a low-dimensional, dense mathematical representation (latent space). In this space, distance correlates with clinical similarity: patients with shared chronic conditions (eg, arthritis) cluster closely together (high similarity), while remaining distinct from patients with unrelated phenotypes, such as acute fractures or healthy controls. This illustrates that “embedding” refers to a learned feature space, not anatomical joints. EHR: electronic health record.

One intuitive and transparent approach is neighborhood-based reasoning (Figure 4). This method compares a patient’s embedding to others in the latent space, assuming proximity indicates clinical similarity. By identifying nearest neighbors, the system can analyze prior treatments and outcomes. If a therapy was successful among close neighbors, it might be recommended for the current patient. This local inference, like case-based reasoning, offers a familiar paradigm for clinicians.

The utility of neighborhood-based reasoning depends on how “similarity” is defined and measured within the multimodal representational space [34]. In our framework, clinical similarity is quantified using mathematical distance metrics, such as cosine similarity or Euclidean distance, applied to the learned latent embeddings. Proximity in this shared space indicates that 2 patients share deeply aligned multimodal profiles, even if their superficial characteristics differ.

To make this actionable, these distances can be mapped to specific clinical metrics: for instance, a newly diagnosed patient might be found to reside in a “neighborhood” where 80% of historical cases responded favorably to tumor necrosis factor inhibitors or shared a high likelihood of flares within 6 months. By identifying these nearest neighbors, clinicians can transition from abstract data points to concrete comparative outcomes, such as identifying shared phenotypic trajectories or similar risks for specific comorbidities. This transforms the latent space from a purely computational construct into a clinically actionable map that informs treatment selection based on the proven successes and failures of “look-alike” patients.

Alternatively, a predictive modeling approach can be employed by training downstream classifiers or regressors directly on the embedding space. Here, embeddings serve as input features for models predicting specific clinical targets, such as disease progression, treatment efficacy, or adverse event risk. This method leverages the compact, information-rich nature of embeddings, which are often better suited for learning than high-dimensional raw data. For example, a simple feedforward neural network, logistic regression, or gradient-boosted trees could predict treatment response likelihood, enabling scalable and fast clinical decision support.

**Figure 5.**
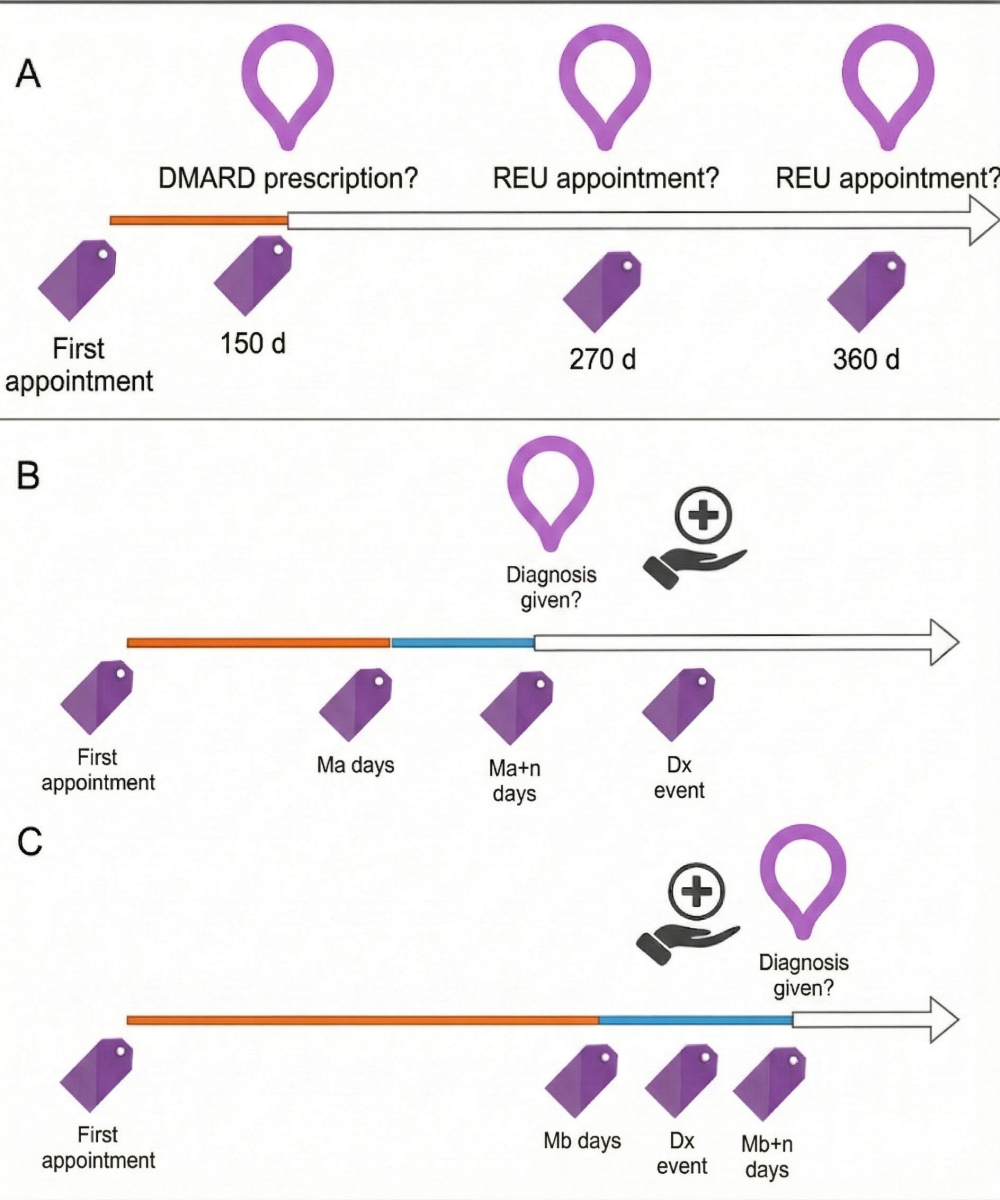
Strategies for clinical event forecasting within the Rheumatic Digital Twin. (A) Fixed-window prediction: illustration of a static forecasting task where patient data gathered during a fixed initial window (eg, the first 150 d) is used to predict specific downstream events, such as a future rheumatic (REU) appointment or a disease-modifying antirheumatic drug (DMARD) prescription at set time points (270 or 360 d). (B and C) Iterative dynamic prediction: illustration of a sliding-window approach where predictions are updated continuously as the patient journey evolves. (B) In the early phase, data available up to point Ma are used to predict a diagnosis within the next n days; the probability is low as the event lies outside the prediction window. (C) As the observation window extends to Mb, the specific diagnosis event falls within the prediction horizon (Mb + n days), resulting in a higher probability forecast. Dx: diagnosis; Ma: earlier observation time point; Mb: later observation time point.

The MDT system can also be used for more specific, yet critical, tasks. It enables forecasting key clinical events, such as predicting the likelihood or timing of significant medical interventions, specialist visits, or treatment changes at future points (eg, 150, 270, 360 d) in the patient’s care pathway (Figure 5A). The MDT system also facilitates early disease diagnosis prediction, allowing for the estimation of a specific diagnosis’s probability within a future window based on a patient’s historical data, which is crucial for timely intervention (Figure 5B,C).

In practice, these approaches are not mutually exclusive. A robust DT system could combine them, using neighbor-based reasoning for transparency, predictive models for speed, generative models for scenario planning, and active learning for uncertainty management. Together, they provide a rich toolkit for turning multimodal embeddings into personalized, data-driven clinical actions.

### Case Vignette: Dynamic Stratification in RA

To illustrate the practical utility of the RDT, consider 2 hypothetical patients, “Patient A” and “Patient B,” both presenting with early RA. At baseline, both patients appear clinically identical: they are of similar age, have high disease activity (Disease Activity Score-28 >5.1), and test positive for anti–cyclic citrullinated peptide serology. Under current standard-of-care guidelines, both are viewed as “high-risk” candidates and are prescribed methotrexate as a first-line therapy. However, the RDT would resolve the underlying heterogeneity by ingesting their full multimodal profiles, utilizing *ICD-10* billing codes, longitudinal lab values, and unstructured doctor notes from previous consultations.

While their structured billing codes and diagnosis labels are identical, the joint embedding layer detects subtle, latent differences. Patient A’s embedding is heavily influenced by specific phrases in the doctor’s notes regarding extra-articular manifestations and a pattern of escalating inflammatory lab values, whereas Patient B’s embedding aligns with standard seropositive profiles. Consequently, the RDT projects them into different “neighborhoods” of the latent space despite their superficial similarity. The model maps Patient B to a cluster of historical “methotrexate responders,” validating the standard course of action where the patient proceeds to achieve remission on methotrexate alone. Conversely, the model maps Patient A to a “refractory” cluster, where nearest neighbors historically failed methotrexate and suffered early joint erosion. Instead of waiting 6 months to confirm treatment failure via a physical flare, the RDT triggers an early alert for Patient A, highlighting the divergence from Patient B’s trajectory and supporting a clinical decision to switch disease-modifying antirheumatic drugs (eg, to a targeted biologic) earlier in the care pathway. This comparative analysis demonstrates how the RDT resolves heterogeneity that is invisible to snapshot-based assessments, ensuring Patient A receives aggressive intervention, while Patient B avoids unnecessary escalation.

## Discussion

### Key Contributions

To deliver on the promise of precision medicine, we require tools that can identify meaningful disease subgroups and provide treatment guidance with minimal trial and error. The necessary multimodal patient data, ranging from clinical notes and wet lab diagnostics to imaging and genomic profiling, often exists but is siloed and underused. The modular MDT architecture presented here offers a flexible and scalable approach for harmonizing, modeling, and learning from this diverse data landscape.

The central contribution of this work is the proposal of a modular, multimodal DT architecture that is designed to be robust and clinically relevant. By dividing the learning task into modality-specific foundation models, each trained on a particular data type, the architecture allows for independent development and validation. This modularity enables model optimization for each data type, supports gradual system expansion as new data streams become available, and facilitates cross-institutional collaboration. A major strength is the architecture’s ability to operate with missing data modalities. Embeddings can be created from available modalities, allowing predictions and patient similarity calculations to be made even when data is incomplete. The embedding neighborhood around a patient enables case-based reasoning, providing a human-interpretable link between new and historical cases and offering immediate clinical insight. The architecture also supports incremental learning and dynamic model updates. As more patient data become available, embeddings can be recomputed without retraining the entire system. This adaptability is crucial for real-world deployment. Additionally, it improves interpretability by allowing clinicians to trace recommendations back to similar historical cases. Finally, by structuring predictions around multimodal embeddings, the architecture is compatible with recent advances in large language models. These large language models could further enrich the system by supporting natural language queries, summarizing patient records, or generating clinical hypotheses.

Comparative analysis with existing frameworks: to contextualize the novelty of the RDT, it is necessary to contrast it with existing machine learning architectures commonly used in medical informatics. While standard approaches such as unimodal time-series models (eg, RNNs/long short-term memories) or early fusion static models have proven useful for specific tasks, they face significant limitations when applied to the heterogeneous and fragmented nature of RD data. As summarized in Table 1, the RDT distinguishes itself through its intermediate fusion strategy and Transformer-based backbone. Unlike early fusion models that require complete data vectors (often necessitating artificial imputation) or late fusion ensembles that fail to capture feature interactions, the RDT leverages joint embeddings to maintain high resilience against missing data while preserving cross-modal learning.

**Table 1.** Comparative analysis of the proposed Rheumatic Digital Twin framework vs traditional machine learning architectures.

Feature	Unimodal time-series (RNN^a^/LSTM^b^)	Early fusion static models	Late fusion ensembles	Proposed RDT^c^ (intermediate fusion)
Data integration	Single modality only (eg, labs)	Concatenates raw inputs at start	Averages predictions at end	Fuses learned embeddings in latent space
Temporal handling	Good, but struggles with irregular gaps	None (static snapshots)	Limited (varies by submodel)	High (Transformers+Time-delta tokens)
Missing data	Low resilience (gaps break sequence)	Low resilience (needs full vector)	High resilience	High resilience (via attention masking)
Cross-modal learning	None	High (but brittle)	Low (no feature interaction)	High (via joint embedding alignment)
Scalability	Low (hard to add new data types)	Low (must retrain entire model)	High	High (modular components)

a RNN: recurrent neural network.

b LSTM: long short-term memory.

c RDT: Rheumatic Digital Twin.

### Key Challenges

Implementing this architecture in practice involves overcoming several fundamental challenges. First, multimodal data integration is difficult because health care data is heterogeneous in structure, format, and semantics. Harmonizing data across institutions also introduces variation and potential bias. A related challenge is embedding coherence. Because each modality is embedded independently, there is a risk of losing crucial cross-modal interactions. Future work may need to move toward joint embedding models or employ alignment techniques to preserve these dependencies.

It is important to note that while we discuss federated learning (FL) as a promising architecture for privacy preservation, it represents only one potential pathway for implementation among other centralized or hybrid approaches. In scenarios where FL is adopted, our framework assumes that paired samples (eg, imaging and reports) for a given patient are colocated on the same node. This assumption facilitates local contrastive learning by ensuring simultaneous access to paired modalities during the optimization of local objectives. However, this setup shifts the technical challenge toward global alignment: while local nodes can align their own modalities effectively, ensuring that the learned latent spaces remain consistent across heterogeneous nodes (non–independent and identically distributed data) without centralized data sharing requires robust aggregation strategies to prevent model divergence.

Validation is another substantial hurdle. A DT system that integrates multiple types of predictions and use cases defies easy benchmarking. Furthermore, the MDT’s influence on patient care can create feedback loops that shift data distribution, requiring continuous evaluation and drift detection [35]. Building gold-standard validation datasets that reflect real clinical variation remains an open challenge. Finally, methodological challenges are inherent to real-world health care data. Noise, missingness, and systemic bias are pervasive and can compromise model quality [36]. Correcting these issues requires statistical techniques and continual clinician involvement [37].

While the reliance on foundation models offers significant predictive power, implementing such an architecture in practice involves overcoming substantial computational and practical hurdles. Developing true foundation models typically requires massive data volumes and high-performance computing resources, which can be prohibitively expensive for individual academic medical centers.

Our modular architecture specifically mitigates these challenges by separating the learning task into independent, modality-specific streams. Rather than attempting to train a single monolithic model on all data types simultaneously, a task that would require immense memory and synchronized data access, our framework allows for the distributed, parallel training of smaller unimodal models. This approach enables institutions to optimize and validate a text-based model (eg, for clinical notes) or an image-based model independently, significantly reducing the immediate hardware requirements for any single training phase. By leveraging pretrained weights from existing domain-specific models and focusing local resources on fine-tuning and joint embedding, the RDT becomes a computationally feasible solution for real-world clinical environments.

### Key Considerations

Even with technical challenges addressed, several broader considerations remain. Two of the most important considerations are trust and interpretability. Clinicians must understand not just what the system recommends, but why, and they must retain final decision-making authority. Scalability and infrastructure are also critical. The system must integrate with hospital information systems, meet stringent privacy and security requirements, and be usable in time-sensitive clinical workflows [38]. This requires careful design of interfaces and data governance mechanisms. Ethical and regulatory concerns must also be considered [39]. Systems must be designed with safeguards that ensure transparency, fairness, and accountability. Risks, such as reinforcing existing disparities, must be explicitly mitigated. Lastly, there is the question of generalizability. An MDT that performs well in one hospital may not generalize to others without recalibration.

To address the challenge of poor generalizability across institutions, we propose a strategy that leverages the modularity of the RDT through tiered transfer learning. In this framework, modality-specific foundation models are first pretrained on large, multi-institutional datasets to capture broad, generalizable representations of medical data. Because these models are domain-specific rather than site-specific, they can be developed through collaborative efforts, such as, but not limited to, FL and local fine-tuning, which allow for the extraction of global knowledge while maintaining strict data privacy.

It is crucial to distinguish this resource-intensive model creation phase from the subsequent model adoption phase. While the development of a new foundation model requires high-performance computing provided by a research consortium, individual clinical centers can then adopt these globally pretrained foundation models and perform local fine-tuning on the joint embedding layer and task-specific pipelines. This ensures that the computational burden of training from scratch is borne by the collective federation, while local deployment remains computationally feasible for standard hospital infrastructure.

Individual clinical centers can then adopt these global foundation models and perform local fine-tuning on the joint embedding layer and task-specific pipelines. This localized step allows the system to adapt to institution-specific coding nuances, local demographics, and varying clinical practices without requiring the immense resources needed to build a model from scratch. However, this approach is not without risks; we must remain vigilant against “catastrophic forgetting,” where the model loses its generalizable knowledge during local adaptation, and “domain overfitting,” where the DT becomes so attuned to local biases that it loses its predictive utility for outlier patients. Continuous evaluation and drift detection mechanisms are, therefore, essential to ensure that the RDT remains robust as it moves between different health care environments.

Implementation Roadmap: To bridge the gap between proposal and practice, we outline a realistic implementation roadmap centered on 3 pillars of feasibility.

1. Incremental Integration: Institutions can initiate the RDT using common data sources, such as EHR clinical notes and structured laboratory results. More complex and high-cost modalities, such as genomic profiling or advanced imaging, can be integrated into the existing joint embedding layer later without requiring a total system overhaul.
2. Leveraging Foundation Models: The use of pretrained foundation models significantly reduces the local computational burden. By adopting models already trained on massive multimodal datasets (eg, using open-source models like ClinicalBERT for text), clinical centers only need to invest in the relatively low-cost phase of local fine-tuning and joint alignment. In cases where domain-specific models do not yet exist, institutions may participate in FL consortia to distribute the high cost of training a new model; however, once established, the local operational requirement remains limited to efficient fine-tuning rather than the “monolithic” training of entire systems from scratch.
3. Distributed Parallel Training: The modular architecture allows for the parallel processing of different data streams. This distributed approach enables specialized departments (eg, radiology for imaging, clinical chemistry for routine lab values) to maintain and validate their specific foundation models independently, making the overall system more manageable within the existing organizational silos of academic medical centers.

Legal Liability: MDTs raise fundamental questions about responsibility in clinical decision-making. Because MDTs currently function as decision-support tools rather than autonomous agents, existing regulatory frameworks (eg, European Union Medical Device Regulation; Food and Drug Administration Clinical Decision Support guidance) operate under a human-in-the-loop principle: clinicians remain responsible for interpreting MDT outputs within the broader clinical context. At the same time, responsibility is shared with developers, who must ensure model validity, documentation, and ongoing performance, and with health care institutions that govern data quality and safe deployment. MDTs, however, increase the causal complexity of recommendations because predictions arise from a continually updated model instance. When suboptimal outcomes occur, attributing fault across clinician judgment, data provenance, and model design becomes challenging. This underscores the need for rigorous audit trails, version control, and traceability mechanisms and may ultimately require new legal frameworks capable of addressing distributed or tiered liability in hybrid human-artificial intelligence decisions.

Ownership of the MDT: Continually evolving MDTs also challenge traditional notions of data and model ownership. The underlying personal health data remain the legal rights of the patient, whereas the model architecture, algorithms, and proprietary components are typically owned by the developer. Health care institutions, in turn, control the clinical instantiation of the MDT and bear responsibility for its operational use. Because an MDT becomes a patient-specific and dynamically updated derivative of all 3 layers, clear ownership boundaries are difficult to define. A hybrid ownership model is therefore likely to be required, one that distinguishes among rights over data, rights over the computational model, and rights over the instantiated DT used in clinical care. Future governance frameworks will need to delineate these rights explicitly, address portability between institutions, and ensure accountability for the evolving digital representation of the patient.

### Validation Strategies

Given that the RDT is presented here as a prospective framework, establishing a robust validation pipeline is critical for its eventual translation into clinical practice. We propose a multitiered validation strategy designed to evaluate both the technical integrity of the model and its practical utility for rheumatologists.

Technical Evaluation of Embeddings: The quality and information density of the learned latent space will be primarily assessed through self-supervised reconstruction tasks. By measuring how accurately the foundation models can reconstruct original input data, such as specific lab values or clinical text spans, from their latent representations, we can evaluate whether the embeddings effectively capture the essential features of the patient journey. Additionally, the alignment among modalities can be tested using cross-modal retrieval metrics, determining whether a clinical query can accurately retrieve the corresponding imaging or genomic profile in the shared space.

Assessment of Clinical Utility: To confirm clinical usefulness, the model must be validated against objective “gold standards” in rheumatology. We propose benchmarking the RDT’s predictive capabilities on high-stakes tasks, such as forecasting the timing of disease flares, predicting treatment switches (eg, failure of a specific disease-modifying antirheumatic drug), and estimating future disease activity scores (DAS-28).

To rigorously evaluate performance on these tasks, we propose a comprehensive benchmarking strategy. First, to ensure clinical relevance, the model will be validated against established clinical gold standards, such as the European Alliance of Associations for Rheumatology recommendations and human expert consensus. While we acknowledge that human consensus can be subject to interrater variability, it remains the necessary standard for validating utility in fundamental decision-making tasks.

Complementing this, we will employ technical comparisons to isolate architectural benefits. To demonstrate the added value of integrating longitudinal, multimodal data over standard clinical practice, predictions will be compared against traditional “snapshot” baselines (eg, logistic regression based on the most recent clinical assessment). Finally, to isolate the specific performance gains attributable to the proposed Transformer architecture, we will compare the RDT against established longitudinal methods, such as RNNs or long short-term memories or gradient-boosted trees (eg, eXtreme Gradient Boosting) trained on aggregated historical features.

Interpretability as Validation: Finally, we propose “neighborhood-based reasoning” as a qualitative validation method. By examining a patient’s nearest neighbors in the embedding space, clinicians can verify that mathematical proximity correlates with established medical knowledge. For instance, validation would involve confirming that “neighboring” patients in the DT space historically shared similar inflammatory endotypes or biological responses to specific therapies. This step ensures the model identifies meaningful medical patterns rather than spurious data correlations, providing a transparent and verifiable basis for clinical decision-making.

However, it is important to acknowledge that, while the retrieval of historical neighbors offers clinical transparency, the similarity metric itself is derived from high-dimensional, nonlinear embeddings. Therefore, validation must confirm that mathematical proximity in this latent space consistently maps to clinically interpretable features, thereby establishing a verifiable, if not fully transparent, basis for decision-making.

## Concluding Remarks

In conclusion, the health care system is undergoing rapid and pervasive digitization, resulting in an unprecedented availability of data across all stages of care. This data ubiquity creates new opportunities for purely data-driven approaches, such as the hereby-proposed RDT system, to support critical clinical tasks, including disease subtyping and therapy referral. As ongoing technological advances continue to expand our capacities for data acquisition, exchange, storage, and computation, we believe that such DT-based frameworks will play an increasingly central role in enabling more precise, personalized, and effective care, particularly for intrinsically heterogeneous clinical application fields such as rheumatologic care.

## Abbreviations

BERT: Bidirectional Encoder Representations from Transformers
DT: digital twin
EHR: electronic health record
FDA: Food and Drug Administration
FL: federated learning
*ICD-10*: International Classification of Diseases, 10th Revision
MDT: medical digital twin
RA: rheumatoid arthritis
RD: rheumatic disease
RDT: Rheumatic Digital Twin
RNN: recurrent neural network
SPIDERR: Stratification of Patients using advanced Integrative modeling of Data for the Rheumatic Diseases
